# Is every-day walking in older adults more analogous to dual-task walking or to usual walking? Elucidating the gaps between gait performance in the lab and during 24/7 monitoring

**DOI:** 10.1186/s11556-019-0214-5

**Published:** 2019-05-03

**Authors:** Inbar Hillel, Eran Gazit, Alice Nieuwboer, Laura Avanzino, Lynn Rochester, Andrea Cereatti, Ugo Della Croce, Marcel Olde Rikkert, Bastiaan R. Bloem, Elisa Pelosin, Silvia Del Din, Pieter Ginis, Nir Giladi, Anat Mirelman, Jeffrey M. Hausdorff

**Affiliations:** 1 Center for the Study of Movement, Cognition and Mobility, Neurological Institute, Tel Aviv Sourasky Medical Center, Tel Aviv, Israel; 2 Department of Rehabilitation Sciences, Neuromotor Rehabilitation Research Group, Leuven, KU Belgium; 3 IRCCS San Martino Teaching Hospital, Genoa, Italy; 4 Department of Experimental Medicine, Section of Human Physiology, University of Genova, Genoa, Italy; 5 Institute of Neuroscience, Newcastle University Institute for Ageing, Clinical Ageing Research Unit, Campus for Ageing and Vitality, Newcastle University, Newcastle upon Tyne, UK; 6 The Newcastle upon Tyne Hospitals NHS Foundation Trust, Newcastle upon Tyne, UK; 7 Department of Biomedical Sciences, Bioengineering unit, University of Sassari, Sassari, Italy; 8 Interuniversity Centre of Bioengineering of the Human Neuromusculoskeletal System, Sassari, Italy; 9 Department of Geriatric Medicine, Donders Centre for Medical Neuroscience, Radboudumc Alzheimer Center, Radboud university medical center, Nijmegen, The Netherlands; 10 Department of Neurology, Donders Centre for Medical Neuroscience, Radboud university medical center, Nijmegen, The Netherlands; 11 Sagol School of Neuroscience, Tel Aviv University, Tel Aviv, Israel; 12 Department of Neurology and Neurosurgery, Sackler School of Medicine, Tel Aviv University, Tel Aviv, Israel; 13 Rush Alzheimer’s Disease Center and Department of Orthopaedic Surgery, Rush University Medical Center, Chicago, USA; 14 Department of Physical Therapy, Sackler Faculty of Medicine, Tel Aviv University, Tel Aviv, Israel

**Keywords:** Aging, Mobility, Accelerometer, Dual tasking, Gait, Wearables

## Abstract

**Background:**

The traditional evaluation of gait in the laboratory during structured testing has provided important insights, but is limited by its “snapshot” character and observation in an unnatural environment. Wearables enable monitoring of gait in real-world environments over a week. Initial findings show that in-lab and real-world measures differ. As a step towards better understanding these gaps, we directly compared in-lab usual-walking (UW) and dual-task walking (DTW) to daily-living measures of gait.

**Methods:**

In-lab gait features (e.g., gait speed, step regularity, and stride regularity) derived from UW and DTW were compared to the same gait features during daily-living in 150 elderly fallers (age: 76.5 ± 6.3 years, 37.6% men). In both settings, features were extracted from a lower-back accelerometer. In the real-world setting, subjects were asked to wear the device for 1 week and pre-processing detected 30-s daily-living walking bouts. A histogram of all walking bouts was determined for each walking feature for each subject and then each subject’s typical (percentile 50, median), worst (percentile 10) and the best (percentile 90) values over the week were determined for each feature. Statistics of reliability were assessed using Intra-Class correlations and Bland-Altman plots.

**Results:**

As expected, in-lab gait speed, step regularity, and stride regularity were worse during DTW, compared to UW. In-lab gait speed, step regularity, and stride regularity during UW were significantly higher (i.e., better) than the typical daily-living values (*p* < 0.001) and different (*p* < 0.001) from the worst and best values. DTW values tended to be similar to typical daily-living values (*p* = 0.205, *p* = 0.053, *p* = 0.013 respectively). ICC assessment and Bland-Altman plots indicated that in-lab values do not reliably reflect the daily-walking values.

**Conclusions:**

Gait values measured during relatively long (30-s) daily-living walking bouts are more similar to the corresponding values obtained in the lab during dual-task walking, as compared to usual walking. Still, gait performance during most daily-living walking bouts is worse than that measured during usual and dual-tasking in the lab. The values measured in the lab do not reliably reflect daily-living measures. That is, an older adult’s typical daily-living gait cannot be estimated by simply measuring walking in a structured, laboratory setting.

## Background

Among older adults, gait is one of the keys to functional independence and gait changes are associated with and predictive of numerous adverse health outcomes. These include falls, mobility disability, cognitive decline, dementia, and even mortality [1–3]. Until recently, gait assessments were generally conducted in specialized laboratory facilities, under well-defined, scripted conditions. These tests provided important insight into gait impairments in aging and pathology [4–14]. Cognitive “dual tasking” paradigms were added to enhance short, in-lab testing in an attempt to make the tests more reflective of the many motor-cognitive challenges that occur during every-day ambulation [15–26]. Dual-tasking studies demonstrated that in older adults, people with neurodegenerative diseases, and many other cohorts with impaired walking abilities, gait speed is reduced, gait variability becomes larger, and asymmetry often increases. At the same time, there is increased reliance on cortical function, in particular, the pre-frontal cortex, during walking [27–31]. Dual-task walking abilities and this increased pre-frontal cortex activation have also been related to fall risk [17, 32]. This suggests that during every-day walking, when many secondary tasks provide challenging situations, dual-task walking is commonplace and critical to functional independence. Interestingly, although dual-task walking is presumed to be ubiquitous, an estimate describing how common it is does not yet exist. Thus, its impact on daily-living walking can only be inferred.

Body-worn sensors, also referred to as wearables, now provide an inexpensive opportunity for the continuous monitoring of ambulatory activity in free-living environments [7, 9, 33–39]. The basic elements of gait are similar regardless of where a subject is tested. Yet, like the ambulatory monitoring of real-world arrhythmias and seizures, multi-day, continuous recordings of gait putatively provide metrics that capture the complexities and multiple influences on real-world gait [40] that are not fully reflected when subjects are assessed in the laboratory or clinic. These every-day influences likely include dual and multi-tasking, planning, obstacle negotiation, fatigue, motivation, mind wandering, and mood. Previous studies using wearables to assess daily-living gait have shown the value of such measurements, for example, in predicting future falls among older adults [7, 33–39, 41]. At the same time, a growing body of literature suggests that the values of the gait measures extracted from daily-living differ from those extracted during testing in the laboratory [7, 10, 11, 33, 39, 42–46]. The reasons for this gap are, however, not yet fully clear.

Previous studies have generally compared in-lab usual walking to daily-living measures, however, a direct comparison of in-lab dual tasking gait to daily-living gait has not yet been conducted. We speculate that perhaps the dual- and multi-tasking that occurs during daily-living may contribute to the gaps between in-lab usual walking and daily-living walking. As a step toward better understanding the gaps between in-lab gait and daily-living gait, here we sought to examine the role of dual-tasking. In particular, in the present study, we directly compare in-lab to daily-living gait in older adults with a history of falls in order to elucidate the relationship between the measures obtained in each setting and to gain insight into daily-living walking. We focused on five spatial-temporal gait features that are commonly used to evaluate and characterize in-lab usual walking and in-lab dual-task walking and sought to address the following questions: Is daily-living gait comparable to usual walking, as measured in the laboratory? Stated alternatively, is daily-living gait, which typically takes place in a complex, cognitively challenging environment, more similar to dual-task walking as measured in the laboratory? About how much of daily-living walking is worse than in-lab dual-task walking?

## Methods

### Participants

The present analysis is based on the baseline assessment of subjects who participated in V-TIME, a multi-center (5 clinical sites), randomized controlled trial designed to reduce fall rates in older adults [47]. Briefly, individuals were enrolled if they were aged 60–90 years, on stable medications for the past month, able to walk for at least 5 min unassisted, and had at least 2 falls in the previous 6 months. Individuals were excluded if they had psychiatric comorbidity (e.g., major depressive disorder as in accordance with DSM IV criteria); history of stroke, traumatic brain injury, or other neurological disorders (not including mild cognitive impairment); acute lower back or lower extremity pain; peripheral neuropathy; rheumatic and orthopedic diseases; or a clinical diagnosis of dementia or severe cognitive impairment (Mini Mental State Exam score < 21). Subjects were characterized by age, gender, body-mass-index, and years of education. In addition, the Montreal Cognitive Assessment (MOCA) evaluated general cognitive function [48] (best possible score 30), the SF-36 assessed general health and physical function [49], and the Falls Efficacy Scale-International (FES-I) evaluated fear of falling (best and worst possible scores 16 and 64, respectively) [50]. The Short Physical Performance Battery (SPPB) (best possible score 12) [51], Mini-Balance Evaluation Systems Test (MINI-BEST) (best possible score 32) [52], and the Four Square Step Test (FSST) [53] assessed multiple aspects of balance, gait, and mobility in the lab.

### In-lab assessment of gait

Participants walked back and forth in a well-lit, 15-m long corridor for 1 min under two walking conditions: (1) preferred, usual walking speed and (2) dual-task walking, i.e., while serially subtracting 3s from a predefined 3-digit number while walking, with no explicit task prioritization. The testing order was fixed. To quantify gait, a lightweight body-fixed sensor (Opal APDM, Portland, Oregon) was attached with a belt to the lower back (lumbar vertebrae 4–5). The sensor includes a tri-axial accelerometer, gyroscope, and magnetometer (unit weight 22 g; unit size 48.5 mm × 36.5 mm × 13.5 mm; 128 Hz sampling rate).

### Daily-living assessment of gait

At the end of the laboratory testing session, participants were asked to wear a tri-axial accelerometer (Axivity AX3, York, UK; dimensions: 23.0 × 32.5 × 7.6 mm; weight: 11 g; 100 Hz sampling rate) for one week. The device was held in place with skin tape to the lower back (lumbar vertebrae 4–5). The participants were instructed to leave the device on throughout the week and to continue their daily activities as usual and not to change their routine. Upon completion of the recording, participants removed the device and mailed it back to one of the study sites for data processing.

### Data processing and analysis of gait

**Fig. 1:**
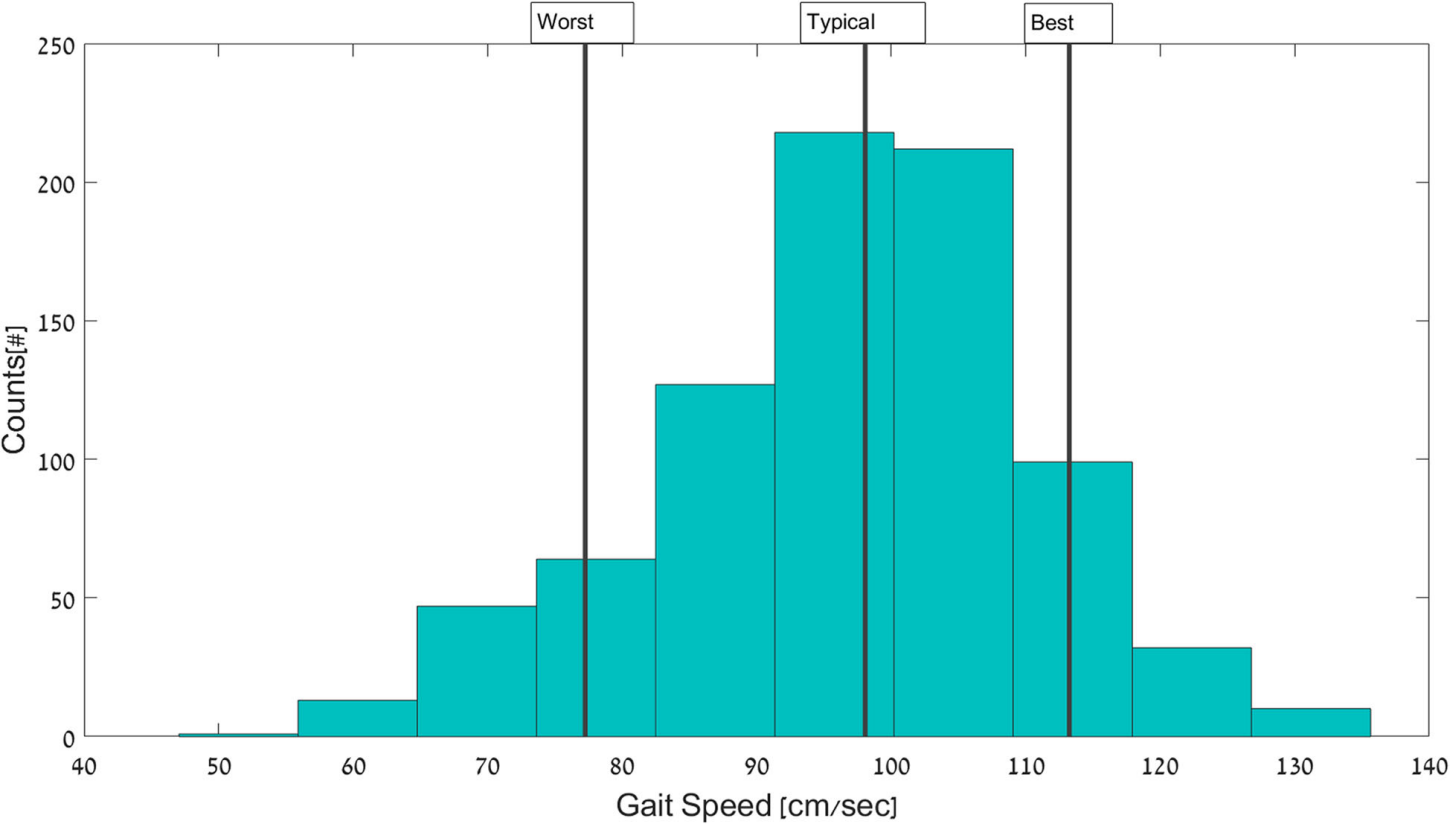
An example histogram from one subject of the values of gait speed obtained during 30-s walking bouts across the week during the daily-living recording. The subject’s typical (50%) gait speed was 98 cm/sec, the worst (10%) was 77 cm/sec and the best (90%) was 113 cm/sec. The use of descriptors “worst” and “best” is according to in-lab terminology where higher = better and lower = worst. These labels may not be appropriate when they are applied to some daily-living conditions (e.g., when walking on a wet, slippery surface, a slower gait speed and a shorter step length may actually be the most appropriate behavior and not the “worst” behavior)

The data analysis of the daily-living recordings included two stages: 1) Detection of all 30-s walking bouts [58]; and 2) Determination of the gait features in each bout, using the same algorithms as those used for in-lab testing. We focused on 30-s walking bouts, as this relatively long length most likely reflects purposeful, steady-state walking assessed in the lab and because relatively long walking episodes are more relevant for assessing walking quality [11, 34, 38, 39, 54]. The outcome measures for both in-lab and daily-living included step time, step length, gait speed, fundamental spatial-temporal gait measures, and were determined as previously described [55–58]. We also assessed step regularity, a measure of gait asymmetry (lower values reflect greater asymmetry) and stride regularity, a measure of the consistency of the walking pattern (higher values reflect greater stride-to-stride consistency and lower values reflect greater stride-to-stride variability) [59]. For each subject and for each feature, a histogram based on the value in all 30-s walking bouts was determined and then from this, each subject’s typical (percentile 50, median), worst (percentile 10) and the best (percentile 90) values over the week were determined (see Fig. 1 for an example). It should be noted that the terms “worst” and “best” are based on labels and interpretations that are usually applied to gait testing in the lab, however, it is not yet fully clear how to apply these terms to measures taken in daily life. In parallel, the outcome measures were extracted for laboratory walking bouts after removing turns and the first and last 3 steps of every walking bout to minimize start (i.e., acceleration and end (i.e., deceleration) effects [60]. Finally, we compared the features extracted from daily-living walking bouts to the in-lab usual and dual-task walking.

### Statistical analyses

Statistical analyses were carried out using SPSS v25 (SPSS Inc., Chicago, IL). To obtain an accurate assessment of daily-living walking, only subjects who had more than 3 days of data were included in the analyses [11]; thus 150 out of a possible 164 subjects were analyzed. Descriptive statistics (means and SD) were calculated for gait and subject characteristics. Outliers, defined as values more than three times the interquartile range, were identified and removed. Paired t-tests were used to examine the relationship between each subject’s typical, best, and worst daily-living walking bouts, on the one hand, and each subject’s in-lab usual walking and dual-task walking, on the other hand. To minimize the effects of multiple comparisons, *p* values < 0.001 were considered as significantly different. Statistics of reliability were assessed using Intraclass Correlation Coefficients (ICC_2, 1_) (two way mixed, absolute, single measures). ICCs values lower than 0.50 indicate poor reliability, values between 0.50 and 0.75 indicate moderate reliability, values between 0.75 and 0.90 indicate good reliability and values greater than 0.90 indicate excellent reliability [61].

## Results

**Fig. 2:**
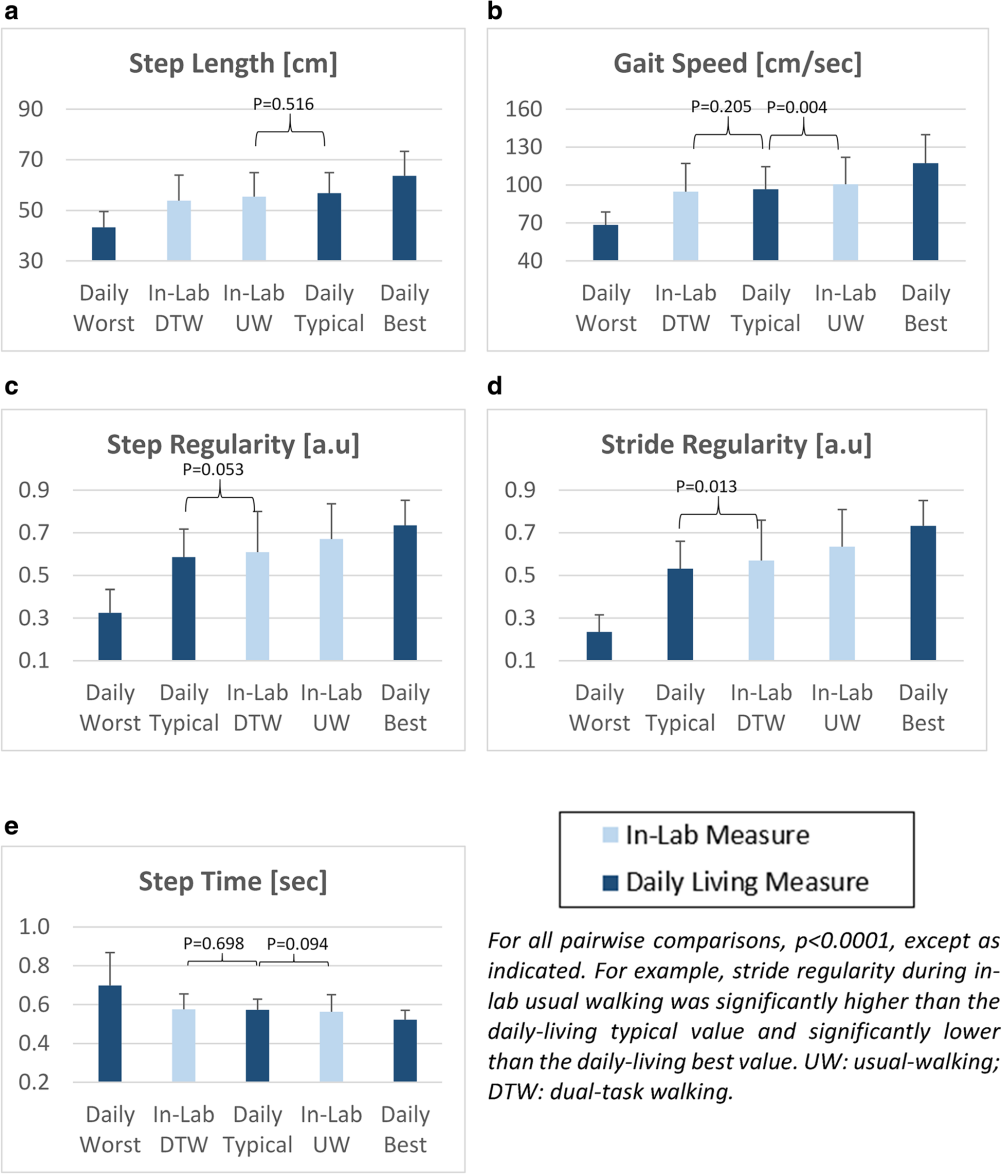
In-lab usual-walking and in-lab dual-task walking compared to daily-living walking typical, best and worst gait values of: **a**) step length; **b**) gait speed; **c**) step regularity; **d**) stride regularity and **e**) step time. The light blue bars reflect the in-lab values of usual-walking (UW) and dual-task walking (DTW). The results shown here are based on 30-s walking bouts

Subject characteristics are summarized in Table 1. The subjects had mild to moderate deficits in cognitive function, balance and physical performance. These characteristics are consistent with that expected of older adult fallers. The light-blue bars in Fig. 2 reflect the in-lab measures of usual-walking and dual-task walking. In-lab gait speed during usual walking was 100.5 ± 21.5 cm/s, consistent with that of older adults with mild to moderate impairment. As anticipated, during the in-lab testing, a significant dual-task effect was seen. For example, in-lab dual-tasking gait speed was lowered (*p* < 0.0001) to 94.7 ± 22.2 cm/s. During in-lab dual-tasking, step time did not change significantly (*p* = 0.146), however, step length, step regularity, and stride regularity were significantly (*p* < 0.0001) lower (i.e., worse) compared to in-lab usual-walking.

**Table 1:** Subject characteristics*

	(*N* = 150)
Age (yrs)	76.5 ± 6.3
Gender (% men)	37.6
Height [cm]	164 ± 8.83
Education (yrs)	12.8 ± 3.9
Body Mass Index (BMI) (kg/m2)	26.2 ± 4.4
Montreal Cognitive Assessment	24.5 ± 3.6
SF-36 General Health	61.3 ± 18.5
Falls Efficacy Scale – International	28.7 ± 8.3
Mini Best Test of Balance (MiniBest)	21.9 ± 6.1
Four Square Step Test (FSST)	12.4 ± 6.8
Short Physical Performance Battery (SPPB)	9.1 ± 2.3
Number of falls in the previous 6 months	2 (2,7)

*Entries are mean ± SD, median (percentile 10, percentile 90), or % as indicated

Figure 2 summarizes the relationship between the gait values of daily-living worst, typical and best walking bouts and the in-lab measures of usual and dual-task walking. As seen in Fig. 2a, in-lab step length during usual-walking was similar (*p* = 0.516) to the typical daily-living value, higher than the daily-living worst, and lower than the best daily-living values (*p* < 0.0001). In-lab dual-task step length differed from the best, worse, and typical daily-living values (p < 0.0001). Gait speed during in-lab dual-task walking was similar (*p* = 0.205) to daily-living typical walking, while in-lab usual-walking gait speed was significantly different from the best (*p* < 0.0001), worst (*p* < 0.0001), and typical daily-living (*p* = 0.004) values (Fig. 2b). For step regularity and stride regularity, in-lab usual walking differed from the best, worse, and typical daily-living values (*p* < 0.0001), while dual-task walking tended to be similar to typical daily-living values (*p* = 0.053, *p* = 0.013 respectively) (see Fig. 2c and Fig. 2d), parallel to what was seen for gait speed. For step time, in-lab usual walking step time and in-lab dual-task walking values were similar to each other and similar (*p* > 0.094) to the typical daily-living value (see Fig. 2e).

**Fig. 3:**
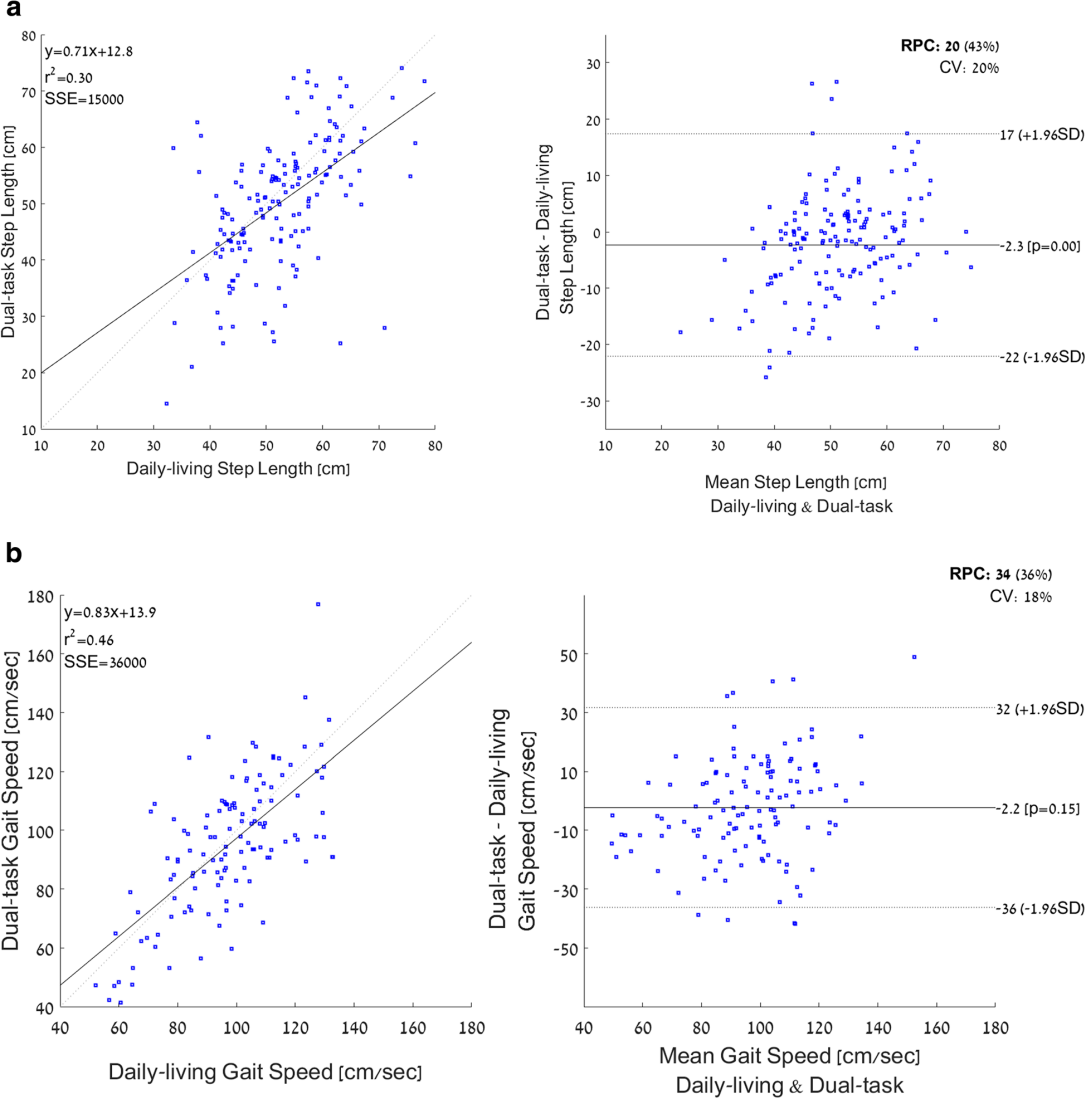
Scatter plots and Bland Altman plots illustrating the relationship between in-lab dual-task step length (**a**) and gait speed (**b**) and the daily-living features observed in 30-s walking bouts. CV: coefficient of variance; RPC: reproducibility coefficient (1.96*SD)

Table 2 summarizes the results of the agreement analyses comparing in-lab and daily-living walking bouts as evaluated using ICC and Pearson’s correlation coefficients for all five gait features. There was good agreement between the values of step length during typical daily-living walking and in-lab usual-walking and moderate agreement with in-lab dual-task walking. For gait speed, step regularity, stride regularity and step time, there was poor to moderate agreement between in-lab values (both usual and dual-task walking) with the typical values obtained during daily-living. Example scatter plots and Bland-Altman plots are shown in Fig. 3 for step length and gait speed, showing the relationship between individual values of in-lab dual tasking values and daily-living values. Many values of step length differed by more than ±5 cm and many values of gait speed differed by than 5 cm/sec (i.e., above the meaningful change difference [57]). This large range around the mean difference is consistent with the relatively high reproducibility coefficients (higher values indicates worse reliability) and the high coefficient of variations (CVs) (higher indicates worse reliability) and suggests that the in-lab values do not reliably reflect the daily-walking values.

**Table 2:**
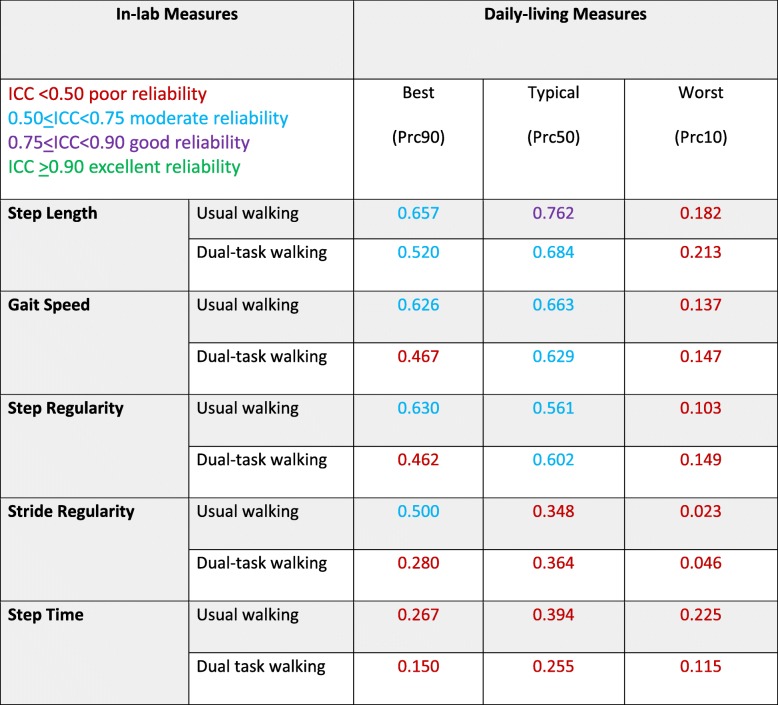
Agreement analyses comparing in-lab features of usual and dual-task walking, on the one hand, and daily-living features, on the other hand, as measured using the intraclass correlation coefficient analyses (two way mixed, absolute, single measure)*

**Fig. 4:**
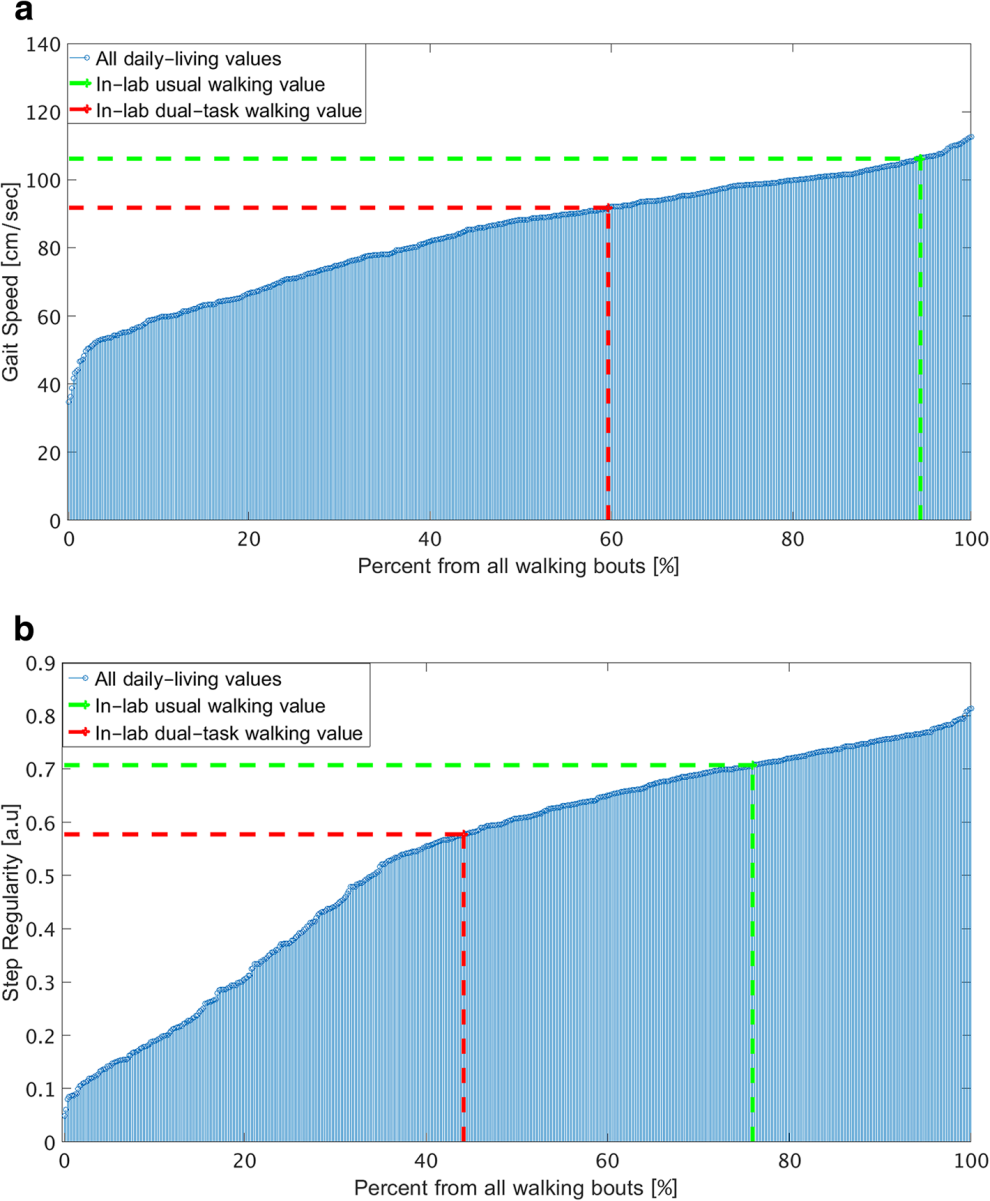
An example of **a**) gait speed and **b**) step regularity for a single subject’s 30-s daily-living walking bouts and his in-lab usual (green line) and dual-task (red line) values

To estimate what percent of each subject’s daily-living walking was worse (e.g., lower) than the corresponding in-lab usual and dual-task walking values, the daily-living values were ranked and determined as a percentile (from 0 to 100, lowest to highest). This allowed us to estimate how the subject’s in-lab values compare (rank) compared to his / her daily-living values. Figure 4 shows an example of gait speed and step regularity daily-living values ranked across all 30-s walking bouts along with the corresponding in-lab usual (green line) and dual-task (red line) values. In this example, in-lab usual walking and dual-tasking gait speed were 106.16 cm/sec and 91.71 cm/sec. This corresponds to the 94 percentile and 60 percentile, respectively, of the daily-living values of gait. In other words, in 94% of all daily-living walking bouts, his gait speed was lower than in-lab usual walking gait speed and in 60% of all daily-living walking bouts, his gait speed was lower than in-lab dual-tasking gait speed.

Table 3 summarizes the percentage of the daily-living walking bouts whose values were worse than those in-lab usual and in-lab dual-task walking values. Averaged across all subjects, daily-living gait speed was lower than in-lab usual walking gait speed in 64% of the daily-living walking bouts and daily-living gait speed was lower than in-lab dual-tasking gait speed in 51% of the walking bouts. To provide an overall summary and general impression, we averaged these percentages across all five gait features. 65% of the daily-living walks were lower than in-lab usual walk and about 55% of the daily-living walks were lower than in-lab dual-task walk (fairly similar to the values for the individual features). In other words, the in-lab measures of gait are better than a large percent of daily-living walking.

**Table 3:** Ranking of in-lab usual walking and in dual-task walking with respect to daily-living 30-s walking bouts*

	In-lab usual walking	In-lab dual-task walking
Step length	53.5 ± 23.1%	45.1 ± 22.9%
Gait speed	63.8 ± 23.3%	50.9 ± 25.4%
Step regularity	73.2 ± 26.6%	64.1 ± 27.5%
Stride regularity	72.3 ± 23.7%	62.1 ± 25.8%
Step time	63.8 ± 25.9%	55.1 ± 29.9%
Average	65.3 ± 24.5%	55.4 ± 26.3%

*Entries are mean ± SD. The values indicate that among 50.9% of all daily-living walking bouts, gait speed was lower than that seen during in-lab dual-task walking, for example

## Discussion

In this cross-sectional study among 150 community-living older adults with mild to moderate deficits in cognitive function, balance and physical performance and multiple falls, we directly compared five commonly used spatial-temporal features of gait quality, as measured in the lab, to the corresponding values obtained during daily-living. When examining relatively long walking bouts (i.e., 30 s), we found three key findings: 1) the group mean values obtained in the lab during dual-task walking are generally similar to the values obtained during daily-living, at least on a group level; however, 2) the specific in-lab measures do not reliably reflect daily-living measures, as seen by the ICC analysis (with the exception of step length in usual walk which is in good agreement with typical daily-living value); 3) more than 50% of the walks in daily-living conditions are worse than the corresponding dual-task values as measured in the laboratory, which is worse than the values obtained during usual-walking in the laboratory.

In general, the comparison between daily-living and in-lab gait features revealed that most gait features obtained during daily-living were closer in value to the dual-task values measured in the laboratory setting (recall Fig. 2). Consistent with this finding, the usual-walking measures obtained in the laboratory tended to be much better than the typical values obtained during daily-living (recall Fig. 2), for most walking bouts (recall Table 3). At the same time, the intraclass correlation coefficients (Table 2) showed poor to good agreement in all the features, suggesting that the values obtained in the lab do not reliably reflect or agree with the same measures obtained during daily-living. Indeed, in the Bland-Altman plot of gait speed (Fig. 3b), many of the data points are above the meaningful change difference of 5 cm/sec [62], illustrating that differences between the in-lab and daily-living values are relatively large. Thus, while dual-tasking in-lab measures are apparently closer to the values determined from daily-living, one is still not a simple mirror image of the other.

In the present study, we focused on the role of one factor that putatively contributes to the gaps between in-lab usual-walking gait and daily-living gait, i.e., dual-tasking. Cognitive-motor and motor-motor dual and multi-tasking are common in daily life, e.g., walking while talking, while using a mobile phone, while carrying a bag, or while watching or negotiating traffic. It is now increasingly recognized that performing two or more tasks simultaneously negatively impacts the gait performance of older adults and that this change is related to adverse health outcomes in aging populations [17, 21, 23, 32, 63, 64]. Thus, dual-tasking assessments have been added to augment short, in-lab testing in an attempt to make them better reflect the many motor-cognitive challenges that occur during every-day ambulation to reveal cognitive compensatory attempts [15–26, 65] and to enhance the ecological relevance of the well-controlled, supervised in-lab testing. Interestingly, we found that gait performance in more than 55% of the daily-living, 30-s long walks are worse than performance observed during controlled testing in the laboratory, even compared with in-lab dual-task values (Table 3 and Fig. 4). This finding implies that in-lab measures of gait, even dual-task walking features, do not provide an accurate reflection of daily-living gait measures. It also suggests that, as a rough approximation, much of daily-living walking apparently involves some factor(s) that makes the performance fall far below that seen during the testing of usual-walking. Given the ubiquitous nature of dual- and multi-tasking in daily-life, we can speculate that these everyday cognitive challenges contribute to the gap between in-lab and daily-living gait. This possibility is consistent with recent findings which showed that cognitive function is more closely correlated to real-world mobility than to laboratory-based mobility [66]. Still, future studies that consider additional factors are needed to further tease out this question. In the meantime, though, it appears that in-lab usual-walking and dual-task walking performance both overestimate much of every-day walking performance.

Other factors likely also play an important role in the gaps between daily-living and in-lab gait. For example, psychological factors like the Hawthorne effect [67] and reverse white coat syndrome [68] are likely to have a positive impact on testing in the lab, with minimal impact on daily-living gait. Factors like mood, depression, and fatigue may negatively impact daily-living gait, more so than on in-lab gait, where study participants may attempt to put on their best effort, regardless of mood and fatigue. In addition to dual-tasking, these factors may have contributed to the gaps that we observed (recall Fig. 2). These ideas have led to the notion that testing in the laboratory represents what the subject can do, i.e., capacity, whereas testing during daily-living represents actual performance, function, and behavior, and not just intrinsic abilities [66, 69, 70]. From this perspective, it may be interesting to compare other types of walking in the laboratory, supervised setting (e.g., fast walking, obstacle negotiation, fatigued walking) to investigate how capacity in these conditions maps to daily-living gait.

Several additional factors to consider are the environment and the nature of the walk. In daily-living, walking may not be along a straight-line. Curved walking has been studied in laboratory settings. It has been shown, for example, that multiple features of gait change during curved walking and when turning [71–76]. In general, during turns and curved walking, gait speed is reduced, asymmetry increases, and the gait pattern becomes more irregular, as compared to straight-line walking; all of these changes are consistent with the finding that typical daily-living gait values of gait speed, step regularity (i.e., symmetry), and stride regularity are all lower than the values seen during testing in the laboratory (recall Fig. 2). In addition, since some of the algorithms used for determining gait quality features assume straight-line, steady-state walking [59, 77], applying them directly to daily-living walks where subjects may walk in a curve, with sharp turns or abrupt changes in speed, might influence the results. Other environmental elements (e.g., an inclined surface, cobblestone sidewalk, lighting) may also contribute to the differences between in-lab and daily-living walking. In this context, it may be helpful to keep in mind that the terms “worst” and “best” were chosen according to gait performance in the lab. In daily life, however, lower and higher values may not necessarily reflect worst and best and the interpretation of the values may depend on the environmental conditions, for example. Perhaps the worst walking bouts during daily-living reflect walking in some of these environmental conditions and are actually an appropriate response (e.g., slower gait speed and shorter step length on a wet, slippery surface). Future work is needed to examine the impact of these additional factors on the gap between in-lab and daily-living gait. Future studies should also examine if and how the present findings apply to other subject groups (e.g., healthy older adults without a history of falls, older adults with widely studied neurological conditions such as Parkinson’s disease) and prospectively evaluate if the time spent in relatively poor walking during daily-living (recall Table 3) changes over time and responds to interventions.

Bout length is also an important consideration. In the current analyses, we controlled for bout length by focusing on 30-s bouts in both the laboratory and daily-living settings. In everyday situations, most relatively long-walks, e.g., 30 s and longer, likely occur outside of the home (i.e., most homes do not have 30-m long paths); in contrast, within the home or office setting, there are many short walking bouts [34]. If steady-state gait and walking performance are the questions of interest, relatively long bouts should be evaluated, as in the present study. At the same time, since much of daily-living gait takes place during very short bouts (< 10 s) [33, 34, 78], these bouts should also be considered when describing all of daily-living functioning.

## Conclusions

In conclusion, the present findings suggest that in-lab measures of gait do not accurately reflect daily-living gait measures. This is the case for in-lab usual-walking and also for in-lab dual-task walking. As noted in the introduction, the assessment of usual-walking and dual-task walking in laboratory settings is valuable, insightful, and clinically relevant, predicting important adverse health events. Nonetheless, the outcomes of our analyses indicate that this snapshot picture of gait does not accurately reflect every-day walking. Using an analogy from cardiology, the present results suggest that just as both the resting (in-lab) ECG and the continuous, daily-living Holter monitoring are informative for assessing and tracking cardiovascular risk, so too, the evaluation of gait based on both in-lab and daily-living testing apparently capture complementary aspects. Still, prospective and additional studies are needed to further demonstrate the utility of these daily-living measures of gait, to better understand what subject characteristics and other factor affect them and the gaps between in-lab and daily-living measures, and to more fully evaluate their potential in the assessment of fall risk, mobility impairment, cognitive decline, and related outcomes that affect many older adults.
